# CapMux: a Snakemake pipeline for early demultiplexing of split-pool scRNA-seq data into sample-resolved outputs

**DOI:** 10.3389/fbinf.2026.1846065

**Published:** 2026-06-12

**Authors:** Denis Baronas

**Affiliations:** Institute of Biotechnology, Life Sciences Center, Vilnius University, Vilnius, Lithuania

**Keywords:** CapSeq, sample demultiplexing, sequencing data processing, single-cell RNA sequencing, split-pool combinatorial barcoding

## Abstract

Single-cell RNA sequencing methods based on split-pool combinatorial barcoding enable high-throughput profiling, yet sample identity is often encoded during early barcoding steps rather than through the library index. Consequently, reads from multiple biological samples remain pooled, complicating per-sample analysis and selective extraction of samples of interest. Here, I present CapMux, a Snakemake-based pipeline for processing split-pool scRNA-seq data from raw sequencing files to sample-resolved outputs. CapMux supports workflows starting from either BCL files or FASTQ files and reconstructs sample identity by integrating sub-library index information with the experiment-specific barcoding plate layout. The pipeline was developed for the CapSeq method but is configurable for related scRNA-seq combinatorial barcoding designs through specification of barcode positions and experimental layout. In a controlled cell line mixing scRNA-seq experiment, CapMux resolved pooled data into outputs for each sample, enabling independent quality control summaries, mapping statistics, count matrices, and downstream visualizations. Runtime benchmarking indicated that secondary demultiplexing step added only a modest computational overhead. Together, these results show that CapMux provides a practical and adaptable framework for recovering sample-level resolution from split-pool scRNA-seq data.

## 1 Introduction

Single-cell sequencing methods have become central to modern molecular biology and biomedicine because they enable the resolution of cellular heterogeneity that is obscured in bulk measurements (Nature Methods, 2013; Baysoy et al., 2023; Vandereyken et al., 2023). Reflecting this impact, single-cell sequencing was named “Method of the Year” in 2013 (Nature Methods, 2013). Since then, the field has expanded rapidly, from transcriptome profiling to a broad and growing repertoire of single-cell and spatial multi-omics approaches that integrate genomic, epigenomic, proteomic, spatial, and other molecular layers at single-cell resolution (Baysoy et al., 2023; Vandereyken et al., 2023).

Among these technologies, single-cell RNA sequencing (scRNA-seq) remains one of the most widely used modalities and is supported by a large ecosystem of commercial and open-source platforms (Jonghe et al., 2024; Conte et al., 2024; Sant et al., 2023; De Simone et al., 2025). Current scRNA-seq methods vary in throughput, transcript coverage, sensitivity, and experimental complexity, but high-throughput approaches are commonly based on either droplet-based compartmentalization or plate-based combinatorial indexing strategies (Conte et al., 2024; Sant et al., 2023; De Simone et al., 2025; Zilionis et al., 2017; Cao et al., 2017; Rosenberg et al., 2018). In both cases, sequencing reads are associated with synthetic cell-identifying barcodes; however, the way sample identity is encoded and later recovered differs substantially between these two families of methods.

In conventional droplet-based methods, a sequencing library is typically prepared for a single experimental sample, and library indexes can therefore be used to separate libraries at an early stage of analysis (Klein et al., 2015; Clark et al., 2023; De Rop et al., 2022; Zheng et al., 2017). By contrast, in plate-based combinatorial barcoding methods, sample identity is often introduced during the first barcoding round (e.g., reverse transcription), after which cells or nuclei are pooled and redistributed across subsequent rounds of barcoding (Rosenberg et al., 2018; Xie et al., 2024; Li and Humphreys, 2022; Cao et al., 2019). As a result, sample identity cannot be resolved from the final sub-library index alone and instead must be reconstructed computationally by integrating sub-library index information with the internal barcode structure.

This design creates a practical bottleneck for downstream analysis. In many split-pool methods, raw sequencing data from multiple samples remain entangled until barcode parsing, alignment, and count-matrix generation have already progressed substantially. Consequently, extracting a single sample of interest, performing per-sample quality control, or reanalyzing only a subset of samples with alternative downstream pipelines becomes unnecessarily cumbersome. The same issue also complicates data reuse from public repositories, where users often must download and process sub-library FASTQ files containing reads from pooled samples even when only a single sample is of interest. Although several processing workflows exist for split-pool scRNA-seq data, they are typically method-specific (Tran et al., 2022; Kuijpers et al., 2024; ScaleRna, 2022), which limits their use as flexible, user-configurable tools for early demultiplexing of sub-libraries into sample-resolved datasets prior to downstream analysis.

Here, I present a bioinformatic pipeline designed to address this gap by enabling early and flexible demultiplexing of split-pool scRNA-seq data at the raw-read level, prior to downstream analysis. The pipeline was initially developed for the CapSeq scRNA-seq method (Baronas et al., 2026), but it was intentionally designed to remain adaptable to future changes in barcode architecture and experimental layout. In addition to supporting CapSeq, the framework can be configured for other similar split-pool combinatorial barcoding designs by specifying barcode positions, barcode counts, unique molecular identifier (UMI) structure, and indexing scheme. To make the pipeline accessible, users provide sample metadata and plate layout information through a simple input file and specify experiment-specific parameters in a configuration file, thereby minimizing the need for custom scripting while supporting diverse experimental designs.

## 2 Results

### 2.1 Pipeline architecture and workflow

**FIGURE 1:**
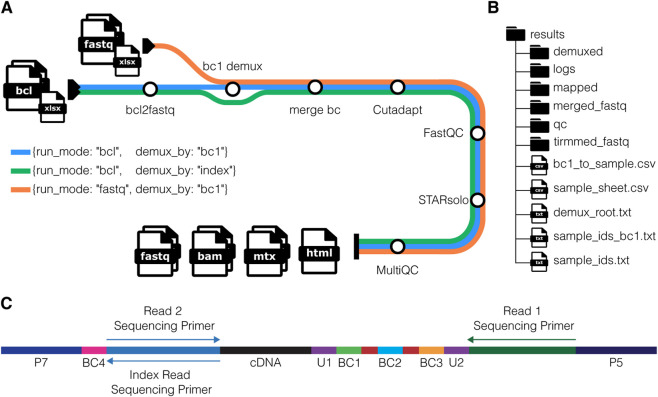
Overview of the CapMux workflow, output structure, and CapSeq library structure. **(A)** Schematic of three CapMux execution regimes. The main workflow, including the secondary demultiplexing step, is shown in blue. In green, only primary index-based demultiplexing is performed. In orange, the workflow starts from FASTQ files. **(B)** Representative structure of the CapMux output directory. **(C)** Structure of CapSeq library (Baronas et al., 2026). The construct includes BC1, BC2, BC3, and BC4 barcode segments, as well as a split UMI composed of U1 and U2 segments. Segments highlighted in red indicate the ligation introduced linker sequences.

The CapMux pipeline can be divided into several sequential steps, as illustrated in Figure 1A. The selected execution regime depends on how the user populates the configuration file and the sample sheet. Specifically, the pipeline can be initiated either from raw Illumina BCL files (run_mode = “bcl”) or from already demultiplexed FASTQ files (run_mode = “fastq”). In the main workflow, in which libraries are further demultiplexed according to the BC1 barcoding plate, the user selects demux_by = “bc1” and provides plate layout in the sample sheet that matches the experimental plate design. In contrast, when the experiment does not contain BC1-defined samples, the user can choose the standard demultiplexing mode, demux_by = “index”, which performs demultiplexing based solely on the sample index sequence.

In split-pool scRNA-seq experiments, the cell barcode (CB) typically consists of multiple barcode segments (for example, BC1-BC2-BC3), which may differ in length and may be separated by linker sequences (Figure 1C). To accommodate this variability, the configuration file allows the user to define the CB structure explicitly by specifying the length of each individual barcode segment. At present, the pipeline supports construction of CBs composed of one to three segments and, when appropriate, one additional barcode segment located in the index read (i7 or i5). The user can also define the unique molecular identifier (UMI) length and specify whether the UMI is contiguous or split into two parts, as in the CapSeq barcoding scheme (Figure 1C). Index files containing barcode sequences must be placed in the assets/barcodes directory, with the barcode sequences in the BC1 barcode index file ordered to match their positions on the physical BC1 barcoding plate, from A1 to H12. Taken together, these settings enable flexible adaptation of the pipeline to different experimental designs while maintaining a standardized processing framework.

**FIGURE 2:**
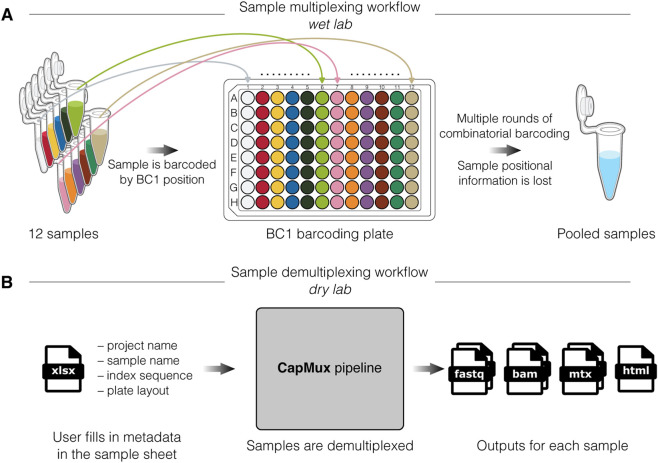
Sample multiplexing and demultiplexing workflows. **(A)** *Wet-lab* sample multiplexing workflow. Sample identity is assigned by BC1 position on the BC1 barcoding plate. After pooling and multiple rounds of combinatorial barcoding, the original sample positional information is lost. **(B)** *Dry-lab* sample demultiplexing workflow. The user provides experiment metadata in the sample sheet, which CapMux uses to reconstruct sample identity and generate sample-resolved outputs, including FASTQ files, BAM files, count matrices, and quality control reports.

In the main CapMux execution regime (Figure 1A, blue), the workflow consists of the following key steps. First, bcl2fastq performs primary demultiplexing by converting raw sequencing data into standard FASTQ files based on the index read. Second, bc1 demux carries out secondary demultiplexing, splitting index-based FASTQ files into sample-specific FASTQ files according to the user-defined BC1 plate layout. Third, merge bc assembles the barcode read by reconstructing the complete CB together with the UMI sequence while removing linker sequences. Next, Cutadapt trims the template switching oligo (TSO) sequence from complementary DNA (cDNA) reads. FastQC is then used for quality control of the FASTQ files, followed by STARsolo, which aligns reads and generates a count matrix for each sample. Finally, MultiQC compiles a structured quality control summary for all demultiplexed samples. Completion of the pipeline yields a structured set of output files within the results directory (Figure 1B). Among these, the principal outputs are demultiplexed FASTQ files, aligned BAM files, gene expression count matrices, and quality control reports generated for each individual sample (Figure 2B).

### 2.2 Sample demultiplexing: resolving sample identity after combinatorial barcoding

As illustrated in Figure 2, sample identity is introduced during the *wet-lab* procedure through the BC1 barcoding plate, but this positional information is lost after pooling and multiple rounds of combinatorial barcoding. CapMux resolves this by applying a secondary demultiplexing step before alignment. In this step, each group of FASTQ files generated for a single index during primary demultiplexing is processed independently and handled in parallel. For each group, the pipeline extracts the BC1 sequence from the user-defined barcode structure, matches it to the sample layout on the BC1 plate derived from the sample sheet, and writes reads into multiple sample-specific intermediate FASTQ files. BC1 matching can be performed either by exact whitelist matching or, when mismatch correction is enabled through the barcode.allow_mismatches parameter, by considering BC1 sequences that differ by a single nucleotide substitution from a whitelist barcode. Reads are assigned to a sample only when the observed or corrected BC1 sequence maps uniquely to one whitelist barcode, while unmatched BC1 sequences are written to undetermined output files. At the same time, the pipeline reconstructs a barcode read by concatenating the configured barcode segments, index sequence, and UMI sequence, thereby generating the paired barcode and cDNA FASTQ outputs required for downstream mapping. After all index-based FASTQ files have been processed, intermediate files belonging to the same sample are merged and compressed in parallel to produce final per-sample FASTQ files for subsequent analysis.

### 2.3 CapMux showcase in a cell line mixing experiment

**FIGURE 3:**
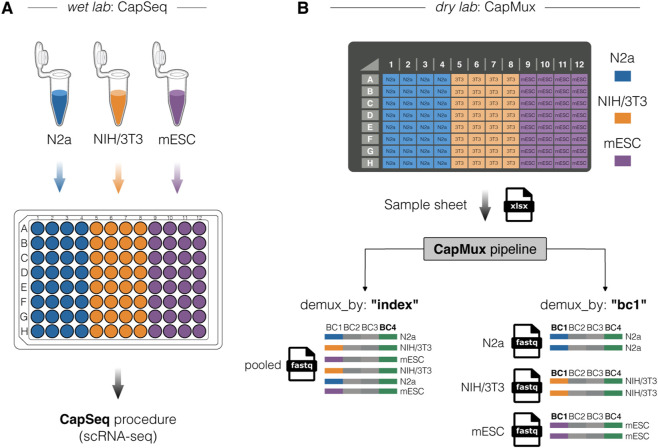
Experimental design of the cell line mixing experiment. **(A)** scRNA-seq library preparation using CapSeq (Baronas et al., 2026). Three mouse cell lines were distributed across the BC1 barcoding plate: N2a cells (blue) in columns 1–4, NIH/3T3 cells (orange) in columns 5–8, and mESCs (purple) in columns 9–12. **(B)** Sample demultiplexing using CapMux. Index-based demultiplexing (demux_by = “index”) produced a single pooled output, whereas BC1-based demultiplexing (demux_by = “bc1”) resolved the experiment into separate outputs for each sample. The assembled barcode structure is also shown, with blue, orange, and purple indicating the BC1 barcode segment corresponding to the sample position on the BC1 barcoding plate, whereas green indicates the sample index sequence provided in the sample sheet and labeled here as BC4.

To demonstrate CapMux in a controlled setting, a CapSeq scRNA-seq experiment was performed using 3 mouse cell lines (Figure 3A). Each cell line was distributed across four BC1 plate columns: Neuro-2a (N2a) cells in columns 1–4, NIH/3T3 cells in columns 5–8, and mouse embryonic stem cells (mESCs) in columns 9–12.

Following sequencing, raw BCL files were processed with CapMux using two execution regimes, both with run_mode = “bcl” but differing in the demux_by parameter (Figure 3B). In the first regime, demux_by = “index”, only primary index-based demultiplexing was performed, resulting in a pooled output. In the second regime, demux_by = “bc1”, the pipeline performed secondary demultiplexing and generated sample-resolved outputs.

**FIGURE 4:**
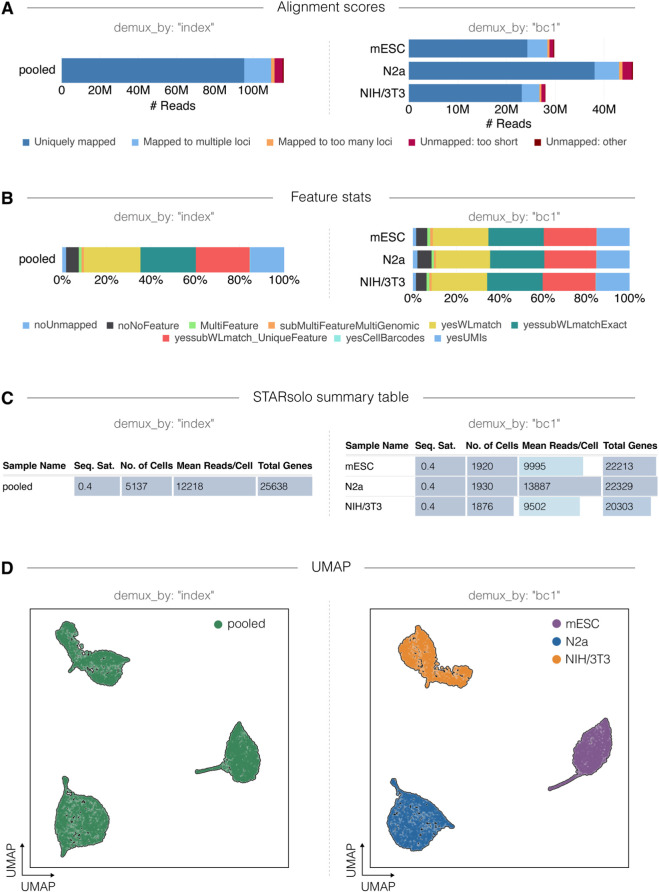
Comparison of CapMux generated results under different demultiplexing regimes. Outputs generated with demux_by = “index” (left) and demux_by = “bc1” (right) are compared for the same cell line mixing experiment. **(A)** Alignment scores, **(B)** feature assignment statistics, and **(C)** summary tables produced by STARsolo. **(D)** UMAP projections. In each case, index-based demultiplexing produced a single pooled output, whereas BC1-based demultiplexing resolved the experiment into separate outputs for each sample.

Comparison of the resulting quality control summaries and UMAP visualizations (Figure 4) highlights the main advantage of secondary demultiplexing with demux_by = “bc1”: sequencing, alignment, and downstream summary statistics were retained separately for each individual sample, whereas under demux_by = “index” these data remained combined and sample-specific differences were masked. In the alignment summaries (Figure 4A) and feature assignment categories (Figure 4B), the demux_by = “index” regime reported only a single combined profile for the pooled dataset, whereas the demux_by = “bc1” regime resolved the data into separate sample-specific profiles for N2a, NIH/3T3, and mESCs. Likewise, in the STARsolo summary tables (Figure 4C), the demux_by = “index” regime produced a single aggregated output, whereas demux_by = “bc1” produced three separate outputs corresponding to the 3 cell lines. This enabled direct comparison of sample-specific metrics, including the number of recovered cells, reads per cell, number of detected genes, and other summary metrics.

The same pattern was also evident in the UMAP projections (Figure 4D). Under demux_by = “index”, all cells remained combined within a single pooled output, whereas secondary demultiplexing with demux_by = “bc1” separated the experiment into three distinct sample-resolved datasets corresponding to the original BC1 plate layout. Together, these results show that CapMux recovers sample-level resolution from pooled CapSeq data, enabling direct comparison of sample-specific differences in read distribution, mapping statistics, feature assignment, and other output metrics in downstream analysis.

### 2.4 Runtime comparison across execution regimes

Workflow wall-clock runtime, measured after prior software environment preparation, differed only modestly across the tested CapMux execution regimes (Table 1; Figure 1A). Under identical input data, pre-created software environments, and fixed high-performance computing (HPC) resource conditions, execution regime A (run_mode = “bcl”, demux_by = “bc1”), which includes the additional secondary demultiplexing step, required only a small increase in runtime compared with execution regime B (run_mode = “bcl”, demux_by = “index”), in which only primary demultiplexing based on the index read is performed. In this dataset, which comprised three samples, the inclusion of secondary demultiplexing increased the mean execution time by approximately 4 min. As expected, the fastest configuration was execution regime C (run_mode = “fastq”, demux_by = “bc1”), because this regime starts from index-demultiplexed FASTQ files and therefore bypasses primary BCL-to-FASTQ conversion and index-based demultiplexing. Overall, these results suggest that the additional secondary demultiplexing step adds only a modest runtime overhead.

**TABLE 1:** Comparison of workflow wall-clock runtime across different CapMux execution regimes. Reported values represent the mean ± SD from three runs performed under identical input and HPC resource settings.

Execution regime	run_mode	demux_by	Runtime
A	bcl	bc1	44 min 51 s ± 1min 56 s
B	bcl	index	40 min 46 s ± 2 min 14 s
C	fastq	bc1	35 min 58 s ± 50 s

To further evaluate performance in a larger sample-multiplexed setting, CapMux was also applied to a previously generated CapSeq dataset (Baronas et al., 2026) containing 12 BC1-defined samples distributed across ten sub-libraries. This larger dataset contained approximately 1 billion reads, compared with approximately 100 million reads in the smaller dataset, corresponding to a 10 times larger input. The same HPC resource conditions were used as for the smaller dataset, and execution regime A (run_mode = “bcl”, demux_by = “bc1”) and execution regime B (run_mode = “bcl”, demux_by = “index”) were evaluated. This analysis showed that the same demultiplexing strategy could be readily scaled without changes to the pipeline logic. As expected, runtime increased with the larger input size: regime A required 388 min 19 s ± 7 min 35 s, whereas regime B required 313 m 58 s ± 20 m 2 s. Thus, the inclusion of BC1-based secondary demultiplexing increased mean runtime by approximately 24%, while enabling the generation of sample-resolved outputs for all 12 samples.

## 3 Discussion

Split-pool combinatorial barcoding methods have become increasingly important in scRNA-seq because they enable profiling of very large numbers of cells in a single experiment while avoiding some of the physical constraints of droplet-based methods. However, the same experimental logic that makes these methods scalable also creates a downstream bioinformatics challenge: samples are often pooled early, whereas sample identity is encoded within the internal barcode structure rather than in the final sequencing index alone. As a result, sample-level resolution is often recovered only late in processing, after substantial computational work has already been completed. The results presented here show that CapMux addresses this bottleneck by enabling early reconstruction of sample identity and generating sample-resolved outputs before alignment and count-matrix generation.

CapMux operates at a different stage of the single-cell analysis workflow than downstream analysis tools such as Seurat (Stuart et al., 2019) or Scanpy (Wolf et al., 2018). These tools are typically used after alignment and count-matrix generation, where sample identity can be inferred, for example, through hashing-based approaches or added manually by assigning cell barcodes to sample labels at the count-matrix stage. In contrast, CapMux operates upstream of these analyses, directly at the BCL or FASTQ processing stage. By combining the sub-library index with the internal barcode structure and experiment-specific plate layout, CapMux generates sample-resolved FASTQ files before alignment and quantification. This automates processing directly from raw sequencing data and allows users to extract samples of interest, perform per-sample quality control, or reanalyze only a subset of samples with selected downstream pipelines.

A central idea of this work is that early secondary demultiplexing improves the interpretability of split-pool scRNA-seq datasets. In the cell line mixing experiment, secondary demultiplexing separated pooled data into outputs corresponding to the original samples, thereby revealing sample-specific differences that were obscured when only primary demultiplexing based on the index read was used. This was evident across several levels of analysis, including summary metrics, alignment statistics, feature assignment profiles, and UMAP visualizations. That distinction is practically important, because sample-resolved outputs represent a more natural unit for biological interpretation, troubleshooting, and comparison across experimental conditions.

Although CapMux was developed for the CapSeq method (Baronas et al., 2026), its design was intentionally made more general. Rather than hard-coding a single barcode architecture, the pipeline allows users to define barcode segments and UMI structure. This makes the framework potentially applicable beyond CapSeq to other split-pool methods that follow a similar barcoding logic. Such flexibility may be particularly useful for the development of new or customized split-pool scRNA-seq methods, as existing method-specific pipelines are optimized for a fixed barcode architecture. For example, commercial workflows such as ScaleRna (ScaleRna, 2022) provide end-to-end processing for ScaleBio single-cell platforms, including demultiplexing, alignment, count-matrix generation, and quality-control reporting. Such workflows are valuable for processing data generated with their intended protocols, but newly developed or modified split-pool designs may require substantial custom scripting or dedicated preprocessing workflows before sample-resolved analysis can be performed. CapMux addresses this by allowing users to define experiment-specific barcode positions, UMI structure, indexing scheme, and sample layout information. Thus, CapMux provides a customizable upstream preprocessing framework that may reduce the need to build a new demultiplexing pipeline for each modified or newly developed split-pool barcoding design.

An additional practical observation is that the benefit of secondary demultiplexing was achieved with only a modest runtime penalty. Under the tested HPC conditions, inclusion of the extra demultiplexing step increased mean runtime by 10% in the smaller dataset, whereas in the dataset that was 10 times larger, mean runtime increased by approximately 24%. Although absolute runtimes will depend on dataset size, hardware, storage performance, and experimental design, the results suggest that early reconstruction of sample identity has a manageable impact on overall workflow runtime.

Taken together, CapMux provides a practical solution for processing split-pool scRNA-seq raw data into sample-resolved FASTQ files and downstream outputs. Future work should test CapMux across a wider range of split-pool methods, expand support for additional barcode architectures, incorporate further functionality such as alternative aligners or quantification tools, expanded quality control reporting, and tighter integration with downstream single-cell analysis workflows. Because the pipeline is implemented in a modular Snakemake framework, these developments should be straightforward to integrate, which may help CapMux remain useful as single-cell methods based on combinatorial barcoding continue to gain popularity.

## 4 Materials and methods

### 4.1 Data processing software

The CapMux workflow is implemented in Snakemake (v9.16.3) (Mölder et al., 2025) and executed with per-rule Conda environments to ensure dependency isolation and reproducibility. Illumina base call conversion and initial demultiplexing were performed with bcl2fastq (v2.19.0). Tabular data processing was handled using custom Python scripts built on pandas (v2.2.3) and openpyxl (v3.1.5). TSO sequence trimming was performed with Cutadapt (v5.0) (Martin, 2011). Read alignment and single-cell quantification were carried out with STAR (v2.7.11b) (Kaminow et al., 2021). Read-level quality control was generated with FastQC (v0.12.1) (FastQC, 2010), and run-level summaries were aggregated into a single report using MultiQC (v1.28) (Ewels et al., 2016). Additional preprocessing in custom scripts relied on standard Unix command-line utilities, including GNU coreutils (v9.5), GNU awk/gawk (v5.3.1), gzip (v1.14), and pigz (v2.8). Custom scripts were run under Python (v3.13.12).

### 4.2 Runtime benchmarking

Pipeline performance was evaluated using end-to-end wall-clock runtime measured at the level of the Slurm batch step, after prior preparation of all required software environments. Snakemake Conda environments were created in a separate preparatory step before benchmarking, and environment creation or package download time was excluded from runtime measurements. Runtime was therefore defined as the elapsed time from initiation of the batch script on the allocated compute node until script termination during workflow execution. For each execution regime, the same input dataset was processed under identical HPC resource allocation conditions (16 CPU cores, 64 GB RAM). Runtime was obtained from Slurm accounting (sacct).

### 4.3 Cell lines

NIH/3T3 (ATCC, CRL-1658) and Neuro-2a (ATCC, CCL-131) cells were grown in Dulbecco’s Modified Eagle Medium (DMEM, Gibco, 61965026) supplemented with 10% fetal bovine serum (FBS) and 1 × penicillin-streptomycin (PS; Gibco, 15140122). Mouse embryonic stem cells (ATCC, CRL-1821, E14TG2a) were cultivated on dishes coated with 0.15% gelatin in DMEM supplemented with 15% FBS, 1 × PS, 0.1 mM sodium pyruvate (Gibco, 11360039), 0.1 mM β -mercaptoethanol (Gibco, 31350010), 1 mM L-alanyl-L-glutamine (Gibco), 1 × Non-Essential Amino Acids (Gibco, 11140050), 1 × 10^3^ U/mL mouse leukemia inhibitory factor (Millipore), 3 μM CHIR99021 (Sigma) and 1 μM PD0325901 (Sigma). All cell lines were grown at 5% ${\text{CO}}_{2}$ and at 37 °C. Cells were collected at roughly 1 × 10^6^cells/mL, transferred into 15 mL conical tubes, pelleted by centrifugation (100–300 × g for 5 min–7 min), and washed twice with ice-cold 1 × Dulbecco’s Phosphate-Buffered Saline (DPBS; Gibco 14190144). Cell number and viability were assessed with a hemocytometer using 0.2% trypan blue staining.

### 4.4 Cell encapsulation and scRNA-seq experiment

Cell encapsulation was performed as previously described Baronas et al. (2026). Briefly, cells at 3 M/mL concentration in 1 × DPBS were mixed with 30% (w/w) dextran (Darna Bio, DB-DEX-2001) at equal volumes, achieving a final concentration of 1.5 M/mL. The stock 10% (w/w) gelatin methacryloyl solution (GelMA; Darna Bio, DB-GMA-2001) was pre-warmed and diluted with 1 × DPBS to the 4% (w/v) working solution. Cell encapsulation was carried out using a 40 μm -deep microfluidic device, with flow rates set to 100 μL/h for the cell suspension containing dextran, 200 μL/h for the GelMA working solution, and 700 μL/h for a surfactant (Darna Bio, DB-SRF-2001). Encapsulations were performed at 26 °C. The remaining protocol steps, including the scRNA-seq experiment, were conducted following the CapSeq procedure (Baronas et al., 2026) without the hairpin ligation step. Sequencing was performed on the Illumina NextSeq 2000 platform using the following run settings: R1, 43 cycles; i7, 6 cycles; and R2, 89 cycles.

### 4.5 Raw sequencing data processing

To generate cell × gene count matrices, the CapMux snakemake pipeline was run under {run_mode: “bcl”, demux_by: “bc1”}, {run_mode: “bcl”, demux_by: “index”}, and {run_mode: “fastq”, demux_by: “bc1”} execution regimes. All other parameters were identical across runs. Barcode segments were defined as follows: bc1: {start: 32, len: 8 }, bc2: {start: 18, len: 10 }, bc3: {start: 5, len: 8 }, umi1: {start: 1, len: 4 }, umi2: {start: 40, len: 4 }. Reads were aligned to the mouse GRCm39 genome (GENCODE M30).

### 4.6 UMAP construction from scRNA-seq data

Single-cell RNA sequencing data was analyzed in Python using Scanpy (v1.11.4) (Wolf et al., 2018). Low-quality cells were filtered out from the cell × gene count matrices by requiring a minimum of 1,000 UMIs/cell and excluding cells with mitochondrial gene count fractions above 20%. UMAP embeddings were generated from normalized and filtered gene expression matrices. Counts were normalized to 10,000 per cell, after which mitochondrial and ribosomal genes were excluded. Additional gene-level filtering was applied to reduce noise: genes detected in fewer than 5 cells and with expression below 10 counts were removed. The data was then log-transformed. Highly variable genes were identified using scanpy.pp.highly_variable_genes (flavor = “seurat_v3”), and the top 150 genes were selected. These genes were z-score scaled, followed by principal component analysis. A k-nearest neighbor graph was constructed using scanpy.pp.neighbors (n_neighbors = 100), and UMAP embeddings were computed with scanpy.tl.umap (min_dist = 0.3).

## Data Availability

The sequencing data generated in this study are publicly available in the Gene Expression Omnibus (GEO) repository under accession number GSE324486. The CapMux pipeline is available at https://github.com/Boyoron/CapMux.
